# Non-Traumatic Carotid Artery Dissection Following an Episode of Orbital Cellulitis: A Case Report

**DOI:** 10.7759/cureus.11175

**Published:** 2020-10-26

**Authors:** Natesh Shivakumar, Rajini Rajagopal, Jonathan H Norris, Pablo Martinez-Devesa

**Affiliations:** 1 Otolaryngology, John Radcliffe Hospital, Oxford University Hospitals NHS Foundation Trust, Oxford, GBR; 2 Ophthalmology, John Radcliffe Hospital, Oxford University Hospitals NHS Foundation Trust, Oxford, GBR

**Keywords:** sinusitis, orbital cellulitis, non-traumatic carotid artery dissection, horner's syndrome

## Abstract

Carotid artery dissection (CAD) is a haemorrhage into the arterial wall disrupting the intimal layers of the vessel. We present a case of a 16-year-old male with a non-traumatic spontaneous CAD. The patient presented with Horner's syndrome following an episode of orbital cellulitis secondary to sinusitis requiring sinus drainage surgery. Subsequent magnetic resonance imaging (MRI) revealed a CAD. The patient was treated with antiplatelet medication.

## Introduction

Carotid artery dissection (CAD) is a haemorrhage into the arterial wall disrupting the intimal layers of the vessel. It can lead to an intramural haematoma which can cause stenosis of the lumen or complete occlusion of the artery leading to a stroke [1]. The clinical features of CAD include neck pain, neurological deficit secondary to cerebral ischaemia, cranial nerve palsies, and Horner's syndrome [2].

While CADs are mostly attributed to trauma to the neck, non-traumatic causes also occur. Predisposing factors include genetic conditions such as Ehlers-Danlos, Marfan syndrome, and anti-trypsin deficiency. Atheroma and pheochromocytoma have also been associated with CAD [1]. Furthermore, there have been reports of infections triggering CAD, in particular respiratory tract infections [3]. Treatment for CAD is often medical with antiplatelets and anticoagulation with the aim of preventing thrombus formation and embolic disease [2].

We present a case of a spontaneous non-traumatic internal carotid artery (ICA) dissection in a patient who presented with Horner's syndrome following an episode of orbital cellulitis requiring surgical drainage.

## Case presentation

**Figure 1:**
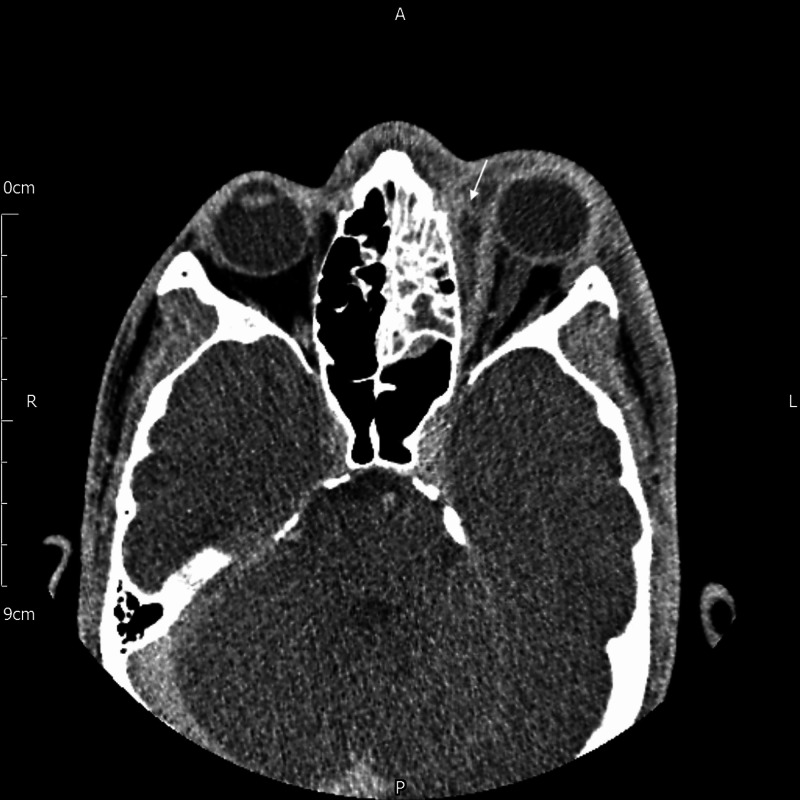
CT scan showing a subperiosteal abscess (white arrow) along the left medial orbital wall along with opacification of the left ethmoid sinus

A 16-year-old male presented to the hospital with a two-day history of frontal headaches, fever, and diplopia. The otolaryngology and ophthalmology team managed him jointly. A computed tomography (CT) head scan revealed features consistent with left ethmoidal sinusitis with a subperiosteal collection within the left medial orbit. Despite management with intravenous (IV) antibiotics, repeat CT showed progression of the abscess (Figure 1). Definitive treatment with functional endoscopic sinus surgery with incision and drainage of the collection was performed.

The patient had no previous history of sinus related disease and was otherwise fit and well. During the follow-up period, the patient clinically improved with a reduction of eyelid swelling and there was no further obstruction of his sinuses. However, he developed a left-sided Horner’s syndrome and was reviewed by ophthalmology. On examination, he had left-sided miosis and ptosis but no anhidrosis. Furthermore, reviewing a previous childhood photograph revealed no anisocoria.

**Figure 2:**
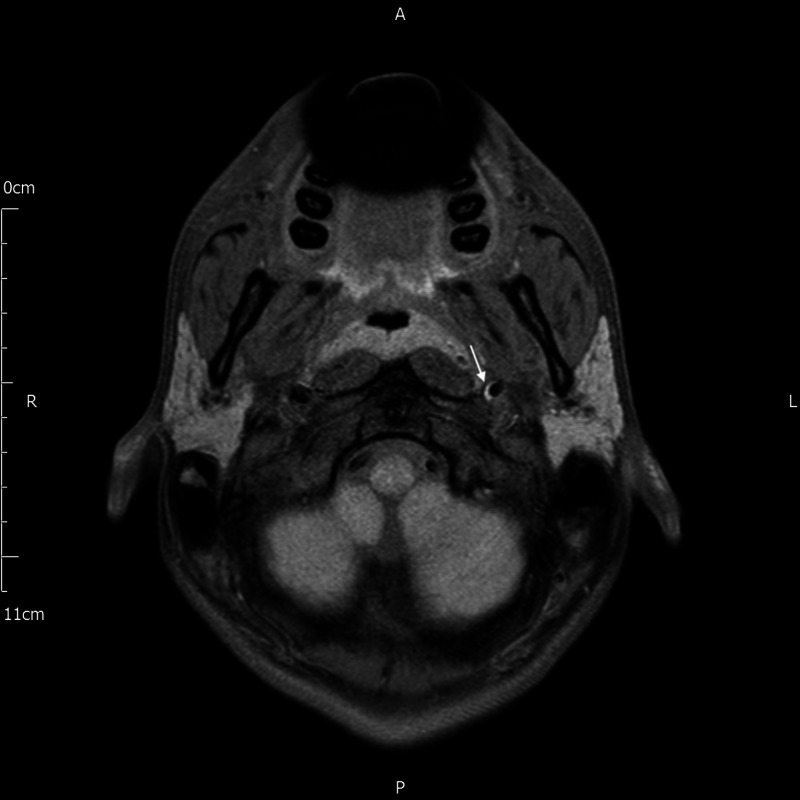
MRI scan with the white arrow highlighting crescent T1 hyperintensity of the medial wall of the left internal carotid artery, suggestive of an intramural haematoma

Subsequent magnetic resonance imaging (MRI) showed a shallow crescent T1 hyperintensity of the medial wall of the cervical part of the left ICA at the skull base. This was suggestive of a small intramural haematoma in keeping with a previous focal dissection (Figure 2). The lumen was regular with no narrowing. MRI brain showed no intracranial pathology and imaging of brachial plexus showed no mass lesions. Furthermore, there was no thyroid or upper mediastinal mass. On reviewing the patient's initial CT scan, there was no clear explanation for his Horner’s syndrome.

Further questioning revealed no history of any trauma to the neck or whiplash, however, he did report an episode of neck stiffness approximately six months prior. He was started on low-dose aspirin, with a plan to perform an interval magnetic resonance angiogram to evaluate any further changes to the haematoma.

## Discussion

Internal CAD is a rare but recognised cause of Horner’s syndrome giving signs of miosis, ptosis, and anhidrosis. Horner’s syndrome results from the disruption of the occulosympathetic pathway which arises in the hypothalamus in the brainstem and runs down the spinal cord to the C8 to the T2 level. It then crosses the apex of the lung and joins the cervical sympathetic chain travelling up the carotid artery into the cavernous sinus, joining the ophthalmic nerve. However, the fibres for sweat function of the face travel along the external carotid artery [4,5].

This case describes Horner’s syndrome resulting from internal CAD. While ptosis and miosis were present, as there was no involvement of the external carotid artery and the sudomotor fibres, there was an absence of anhidrosis in keeping the anatomical route of the occulosympathetic pathway.

The patient had no vascular risk factors, connective tissue disorder, or any history of trauma. A case-control study highlighted an association between recent infection (within four weeks of vascular event) and spontaneous cervical artery dissection (SCAD). It found that acute infection was more common in those with SCAD [6]. Another case report described an internal CAD following a recent episode of a respiratory infection [7].

This case describes an internal CAD following a recent episode of orbital cellulitis requiring sinus drainage surgery. The dissection was of the cervical part of the ICA and anatomically not adjacent to the previous infection site. While the pathophysiology behind the phenomenon of an infection contributing to CAD remains unclear, systemic inflammatory response may play a role. Through increased production of proteases, it could lead to degradation of arterial vessel structure [8]. Furthermore, it is possible that in this case, surgery could have contributed to this systemic inflammatory response along with the infection. There is evidence to suggest inflammatory cells, such as activated T cells and macrophages, are frequently found in the aortas of those with thoracic aortic dissection and aneurysms on histopathologic analysis [8].

## Conclusions

We present a case of spontaneous non-traumatic CAD, presenting with Horner’s syndrome, in a patient with recent orbital cellulitis secondary to sinusitis requiring sinus drainage surgery. Whilst the current published evidence is limited to ascertain with certainty that an association exists, this case highlights the possibility of recent infection being a potential cause or predisposing factor to spontaneous CAD.
